# Improvement of Chronic Rhinosinusitis and Reduction of the Myeloperoxidase-Antineutrophil Cytoplasmic Antibody Titer in a Patient with Eosinophilic Granulomatosis with Polyangiitis by Additional Mepolizumab

**DOI:** 10.1155/2021/5561762

**Published:** 2021-03-29

**Authors:** Shin-ya Tamechika, Shuntaro Isogai, Shinji Maeda, Taio Naniwa, Akio Niimi

**Affiliations:** ^1^Department of Respiratory Medicine Allergy and Clinical Immunology, Nagoya City University Graduate School of Medical Sciences, Nagoya, Japan; ^2^Department of Rheumatology, Nagoya City East Medical Center, Nagoya, Japan

## Abstract

A case of eosinophilic granulomatosis with polyangiitis (EGPA) in which chronic rhinosinusitis (CRS) was improved with a reduction in the myeloperoxidase-antineutrophil cytoplasmic antibody (MPO-ANCA) titer after the addition of mepolizumab is reported. A 55-year-old woman with EGPA receiving prednisolone 5 mg/day developed CRS with increases in the eosinophil count and the MPO-ANCA titer. Although it improved with prednisolone 15 mg/day in addition to mizoribine 150 mg/day, because azathioprine could not be taken orally due to side effects, it relapsed after prednisolone was tapered to 5 mg/day. There was no exacerbation of other vasculitis symptoms such as mononeuropathy multiplex. The patient was treated with additional mepolizumab 300 mg every 4 weeks, which resulted in the improvement of CRS and marked reductions of the eosinophil count and MPO-ANCA titer, and the reduction of prednisolone to 2 mg/day. Furthermore, even after tapering mepolizumab to 200 mg every 4 weeks, her condition remained stable without relapse of EGPA and without increases in the eosinophil count and MPO-ANCA titer. The clinical course of mepolizumab treatment in this patient suggests that the IL5-dependent inflammatory cascade is one of the factors contributing to the increase in MPO-ANCA in EGPA.

## 1. Introduction

Eosinophilic granulomatosis with polyangiitis (EGPA) has been included in the spectrum of antineutrophil cytoplasmic antibody (ANCA)-associated vasculitis [1]. EGPA is characterized by the presence of asthma, as well as blood and tissue eosinophilia [1, 2]; 30–40% of EGPA patients are positive for serum myeloperoxidase (MPO)-ANCA [1, 3–5].

EGPA is treated with glucocorticoids and immunosuppressants; most patients remain dependent on glucocorticoid therapy, and relapses are common [6–9]. Currently, mepolizumab, which is an anti-interleukin (IL)-5 monoclonal antibody, has been approved for the treatment of EGPA. IL-5 is an essential cytokine for eosinophil maturation, activation, and survival [10], and mepolizumab binds to IL-5 and prevents its interaction with its receptor on the eosinophil surface. The MIRRA study showed the efficacy and safety of mepolizumab versus placebo as add-on therapy in participants with relapsing or refractory EGPA, resulting in reductions in the glucocorticoid dose [11]. However, to the best of our knowledge, there have been only a few reports of how mepolizumab affects MPO-ANCA. Thus, the effect of mepolizumab on MPO-ANCA remains unclear. A case of EGPA in which chronic rhinosinusitis (CRS, considered eosinophilic) was improved with a reduction in the MPO-ANCA titer after the addition of mepolizumab is described.

## 2. Case Presentation

**Figure 1:**
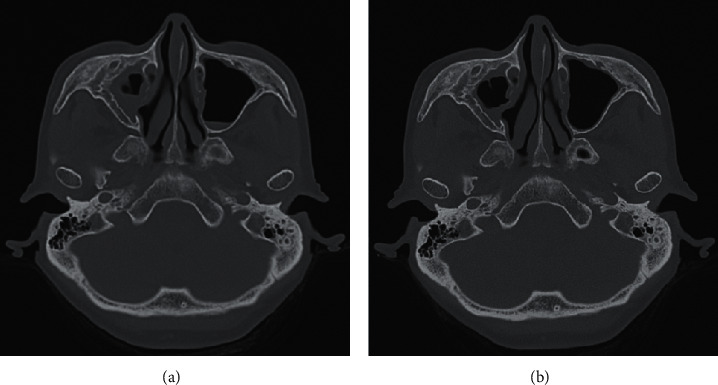
(a) Sinus CT before the administration of mepolizumab. CT shows fluid and mucosal thickening in the maxillary sinuses on both sides, predominantly on the right. (b) Sinus CT in 6 months after the administration of mepolizumab. CT shows a decrease in fluid and mucosal thickening of the maxillary sinuses on both sides.

**Figure 2:**
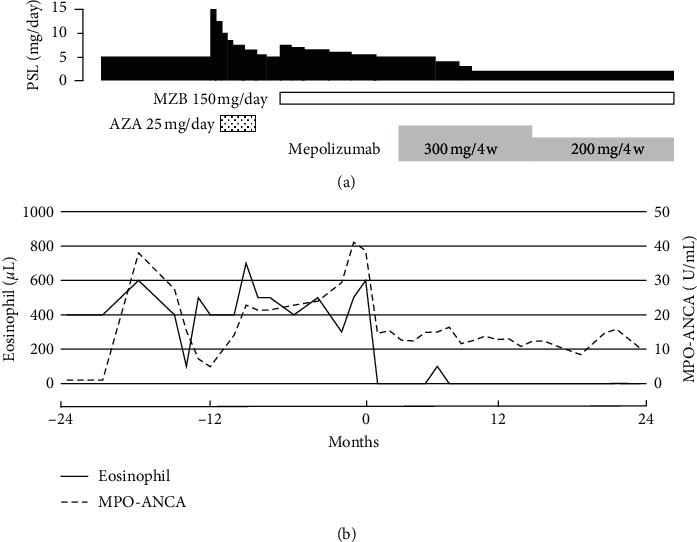
Clinical course. MPO-ANCA: myeloperoxidase-antineutrophil cytoplasmic antibody.

A 55-year-old woman with a history of bronchial asthma was diagnosed with EGPA based on the ACR classification criteria because she had eosinophilia, mononeuropathy multiplex, CRS, palpable purpura, and extravascular eosinophil infiltration confirmed by a skin biopsy [12]. ANCA screening tests showed that MPO-ANCA was positive, antiproteinase-3 ANCA was negative, and indirect immunofluorescence detected perinuclear ANCA staining patterns in neutrophils. No nasal polyps were found, but CRS was considered to be eosinophilic based on a JESREC score of 15 points, which was severe because of the presence of peripheral blood eosinophils of 40.0%, bronchial asthma, and ethmoid shadow ≥ maxillary shadow on CT [13]. She achieved remission with methylprednisolone pulse therapy (1 g/day for 3 days), followed by 40 mg/day of oral prednisolone and intravenous cyclophosphamide, and she was maintained with prednisolone 5 mg/day, along with low-dose macrolide therapy for 5 years. However, EGPA relapsed with an exacerbation of CRS and increases of the eosinophil count and MPO-ANCA titer. Although her condition improved with 15 mg/day of prednisolone in addition to mizoribine 150 mg/day, because azathioprine could not be taken orally due to side effects, it relapsed after prednisolone tapering to 5 mg/day. She suffered from worsening of her rhinorrhea, nasal obstruction, and hyposmia, without exacerbation of bronchial asthma. She had no fever and did not lose weight. There was no exacerbation of other vasculitis symptoms such as mononeuropathy multiplex. Urinalysis showed no proteinuria or hematuria. Her white blood cell count was 5,400/*μ*L with 11.4% eosinophils, hemoglobin was 12.6 g/dL, and the platelet count was 272,000/*μ*L; serum total protein was 7.0 g/dL, with aspartate transferase 15 U/L, alanine transferase 21 U/L, lactate dehydrogenase 192 U/L, blood urea nitrogen 18.0 mg/dL, and creatinine 0.65 mg/dL. Serum C-reactive protein was 0.15 mg/dL, IgG 780 mg/dL, IgA 138 mg/dL, IgM 65 mg/dL, IgE 295 IU/mL, C3 78 mg/dL, C4 24 mg/dL, CH50 55 U/mL, and MPO-ANCA 38.7 U/mL (reference range, <3.5 U/mL). The chest X-ray was normal. Sinus computed tomography (CT) showed maxillary sinusitis and mild ethmoid sinusitis and sphenoid sinusitis, and the Lund–Mackay CT score was 10 points (Figure 1(a)) [14]. Mepolizumab 300 mg subcutaneously every 4 weeks was started without increasing the glucocorticoids. A significant improvement was seen in the clinical symptoms associated with CRS, the eosinophil count, the MPO-ANCA titer, and the sinus radiological findings after 6 months of mepolizumab therapy, and the Lund–Mackay CT score dropped to 3 points (Figure 1(b) and Figure 2) [14]. EGPA did not worsen even after reducing prednisolone to 2 mg/day during continued mepolizumab therapy. Furthermore, even after mepolizumab was tapered to 200 mg every 4 weeks, her condition remained stable without relapse of EGPA and without increases in the eosinophil count and the MPO-ANCA titer (Figure 2).

## 3. Discussion

EGPA, which was known as Churg–Strauss syndrome until 2012, is a multisystem disorder that mainly affects the lungs, heart, nasal passages and sinuses, gastrointestinal tract, and skin [2, 12, 15–17]. Th2 responses and Th1/Th17 responses are considered to be involved in the pathogenesis of EGPA [18]. The prominent Th2 responses upregulate IL-4, IL-13, and IL-5, and the Th1/Th17 responses upregulate interferon (IFN)-*γ*, IL-2, and IL-17, so that eosinophils are activated, they have a long lifespan, and probably cause tissue damage by releasing their granule proteins.

Regarding CRS associated with EGPA, the MIRRA study, a randomized, controlled trial, in 2017 showed mepolizumab reduced the Sino-Nasal Outcome Test-22 (SNOT-22), an indicator of therapeutic effect, and a significant difference between mepolizumab and placebo in the score at weeks 12 and 24 [11, 19, 20]. In this case, the effect of mepolizumab was not evaluated by the SNOT-22 or nasal mucosal biopsy. However, additional mepolizumab without increasing the dose of prednisolone not only improved subjective symptoms but also decreased the Lund–Mackay CT score, along with marked reductions of the eosinophil count and MPO-ANCA titer, which seemed that mepolizumab was effective for CRS.

The MIRRA study already demonstrated that mepolizumab was effective in prolonging disease remission and reducing glucocorticoid use [11]. However, mepolizumab was given in addition to standard care (glucocorticoid treatment, with or without immunosuppressive therapy), and the MPO-ANCA positive rate was only 19% in this study. Since there have been only a few reports examining how mepolizumab affects MPO-ANCA, the effect of mepolizumab on MPO-ANCA is unclear. If EGPA is severe, treatment with high-dose glucocorticoids or immunosuppressants may be required to prevent organ damage. However, since the severity of the present case was classified as localized according to the European Vasculitis Society (EUVAS)-defined disease severity [21], and the Five-Factor Score was 0 points [22], which was a mild case, it was considered that EGPA whose main manifestation was eosinophilic CRS was improved by only mepolizumab without increasing the dose of prednisolone. Recently, Vultaggio et al. reported that subcutaneous administration of 100 mg of mepolizumab every 4 weeks to 18 patients with EGPA showed clinically relevant benefits in exacerbation rates, asthma symptoms, oral corticosteroids, and immunosuppressive use in EGPA patients [23]. Therefore, since only exacerbation of CRS was observed without obvious signs of vasculitis, and no exacerbation occurred even after tapering mepolizumab to 200 mg every 4 weeks in the present case, additional administration of mepolizumab 100 mg every 4 weeks from the beginning may have improved CRS.

Although it is possible that the suppression of eosinophils by mepolizumab affects plasma cells or reduces the expression of April, which inhibits B cell differentiation, leading to MPO-ANCA reduction, the mechanism of MPO-ANCA reduction by IL-5 inhibition is unclear [24–28].

As far as we know, there are two similar reports in addition to the present case. One report involved a patient with EGPA who received mepolizumab that resulted in improvement in eosinophilic otitis media and reduction of MPO-ANCA [29]. The other report involved a patient with EGPA who received benralizumab, an IL-5 receptor antibody, which resulted in improvement in bronchial asthma and reduction of MPO-ANCA [30]. Therefore, although it is still necessary to accumulate cases, it is suggested that suppressing eosinophils may lead to a decrease in MPO-ANCA production in EGPA.

Thus, a case of EGPA in which CRS was improved with a reduction in the MPO-ANCA titer after the addition of mepolizumab was presented. The clinical course of mepolizumab treatment in the present patient suggests that the IL5-dependent inflammatory cascade is one of the factors contributing to the increase in MPO-ANCA titers in EGPA.
